# Direct evidence of an efficient energy transfer pathway from jellyfish carcasses to a commercially important deep-water species

**DOI:** 10.1038/s41598-017-17557-x

**Published:** 2017-12-12

**Authors:** Kathy M. Dunlop, Daniel O. B. Jones, Andrew K. Sweetman

**Affiliations:** 1 Akvaplan-niva, Fram Centre, 9296, Tromsø, Norway; 2 The Lyell Centre for Earth and Marine Science and Technology, Heriot Watt University, Edinburgh, EH14 4AP UK; 3 International Research Institute of Stavanger, Prof. Olav Hanssensvei 15, 4021 Stavanger, Norway; 4 National Oceanography Centre, University of Southampton, Waterfront Campus, European Way, Southampton, SO14 3ZH UK

## Abstract

Here we provide empirical evidence of the presence of an energetic pathway between jellyfish and a commercially important invertebrate species. Evidence of scavenging on jellyfish carcasses by the Norway lobster (*Nephrops norvegicus*) was captured during two deployments of an underwater camera system to 250–287 m depth in Sognefjorden, western Norway. The camera system was baited with two *Periphylla periphylla* (Scyphozoa) carcasses to simulate the transport of jellyfish detritus to the seafloor, hereby known as jelly-falls. *N. norveigus* rapidly located and consumed a large proportion (>50%) of the bait. We estimate that the energy input from jelly-falls may represent a significant contribution to *N. norvegicus* energy demand (0.21 to 10.7 times the energy required for the population of *N. norvegicus* in Sognefjorden). This potentially high energetic contribution from jelly-falls highlights a possible role of gelatinous material in the support of commercial fisheries. Such an energetic pathway between jelly-falls and *N. norvegicus* could become more important with increases in jellyfish blooms in some regions.

## Introduction

Carrion that sinks to the seafloor represents a major energy transfer in the marine environment^1^. Carrion is detected, located and consumed by marine scavengers^2^, enabling the transfer of nutrients and energy back into pelagic and benthic marine food webs^3^. The aggregation of jellyfish carcasses on the seafloor (jelly-falls) are also a source of organic matter input to the benthos^4–6^. Recently, increased frequencies of jellyfish blooms have been observed in a number of regions around the world, including in Norwegian fjords, where *Periphylla periphylla* is now highly abundant^7,8^. A growing body of evidence suggests that the role of gelatinous zooplankton in the biological carbon pump in jellyfish-dominated fjords may be significant, and jelly-falls are actively scavenged here. For example, rapid scavenging on jelly-falls was first shown in Sognefjorden in 2012^9^, with scavenging on jelly-falls being dominated by Atlantic hagfish (*Myxine glutinous*), galatheid crabs (*Munida* sp.) and decapod shrimp (*Pandalus borealis*).

The Norway lobster (*Nephrops norvegicus*) is an economically important commercial species in the Atlantic^10^, and is common in Norwegian coastal regions^11^. In Norway, the revenue from the *N. norvegicus* fishery exceeded 3 million US dollars in 2015^12^. Although the species has been shown to predate and scavenge on a wide range of marine species, primarily decapod crustaceans and fish^13,14^, it has never been observed scavenging on jelly-falls. Here, we describe the first photo-documentation of *N. norvegicus* rapidly scavenging on jellyfish carcasses, and provide empirical evidence of the presence of an energetic pathway between jellyfish populations and this commercially-important species. The role of jelly-falls in the energy budget of *N. norvegicus* is also estimated using values for energy input from jelly-falls and *N. norvegicus* energy demand.

## Material and Methods

Two baited underwater camera (BUC) deployments were made at 250 and 287 m across the outer fjord sill (061° 04.476′ N, 004° 59. 236′ E and 061° 04.087′ N, 005° 00. 378′ E respectively) of Sognefjorden, western Norway in June 2016. Both deployments lasted approximately 10 hours and were conducted during the day (09:10 to 19:07 UTC) and the night (22:25 to 08:02 UTC) respectively. Photographic still images of the jellyfish carcass and attending fauna were taken every 2 minutes by a deep-sea digital single lens reflex camera (Ocean Imaging Systems DSC 24000) system positioned 1.5 meters directly above a square bait plate (0.5 m^2^). For each BUC deployment, the bait plate was baited with two defrosted *P. periphylla* carcasses (~266 g ± 26, mean ± range). The number of scavengers from each species at the bait, the maximum number of scavengers observed at the bait at a single time (Max_N_, a proxy for scavenger abundance) and the time to first scavenger arrival (t_arrival_) were recorded from photographic images from each deployment. The flux of jellyfish material from the water column as jelly-falls (kJ m^−2^ d^−1^) to the seafloor was estimated using mass input data and bomb-calorimetry analysis described in previous studies^5,9^. Jellyfish carrion flux rates were compared to *N. norvegicus* daily energy intake rates (kJ d^−1^) using daily food intake data from a previous study that was adjusted for temperature using Q_10_^14^. Also, estimates of the energy content of *P. periphylla* tissue that were attached to each BUC (kJ g dry weight d^−1^) were calculated based on the mass of jellyfish and bomb calorimetry analysis from^9^.

Data on the density of *N. norvegicus* on the seafloor is required to determine the contribution of jelly-falls to their energy demand. Previous BUC studies have calculated the density of scavengers using the t_arrival_ method^15^. This model works well with abyssal t_arrival_ data sets, where t_arrival_ is generally longer (e.g. >100 minutes) than for datasets collected from shallower depth zones, as highlighted in a previous study^16^. Therefore, *N. norvegicus* seafloor densities in Sognefjorden were based on minimum and maximum *N. norvegicus* densities from other boreal coastal/fjord environments. The data used came from earlier studies in the Firth of Clyde (0.10 to 0.55 individuals m^−2^, ^17^), Scottish sea lochs (Loch Torridon, (0.13 individuals m^−2^, ^18^) and Loch Aline (0.18 individuals m^−2^, ^19^)), the Irish Sea (0.13 to 0.31 individuals m^−2^)^20^), the Kattegat and Skagerrak (0.2 to 0.4 individuals m^−2^, ^11^) and the North Sea (0.09 to 0.73 individuals m^−2^)^21^.

### Data Availability Statement

No restrictions exist on the availability of material and data.

## Results and Discussion

**Figure 1:**
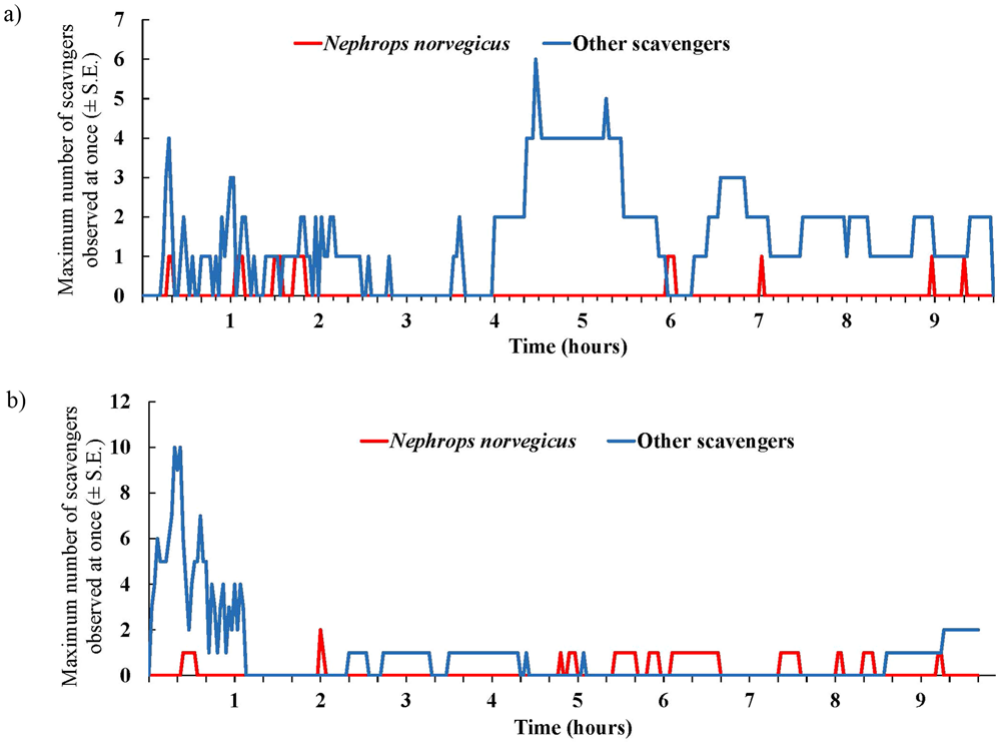
Maximum number of *Nephrops norvegicus* and other scavengers (inc. *Myxine glutinosa*, *Munida* sp. and *Pandalus borealis*) observed in the BUC deployment at (**a**) 250 m (arrival time of BUC on seafloor 07:10 UTC; 19 June 2016) and (**b**) 287 m (arrival time of BUC on seafloor 20:25 UTC; 21 June 2016). Time represents the minutes that elapsed after arrival of the BUC on the seafloor.

**Figure 2:**
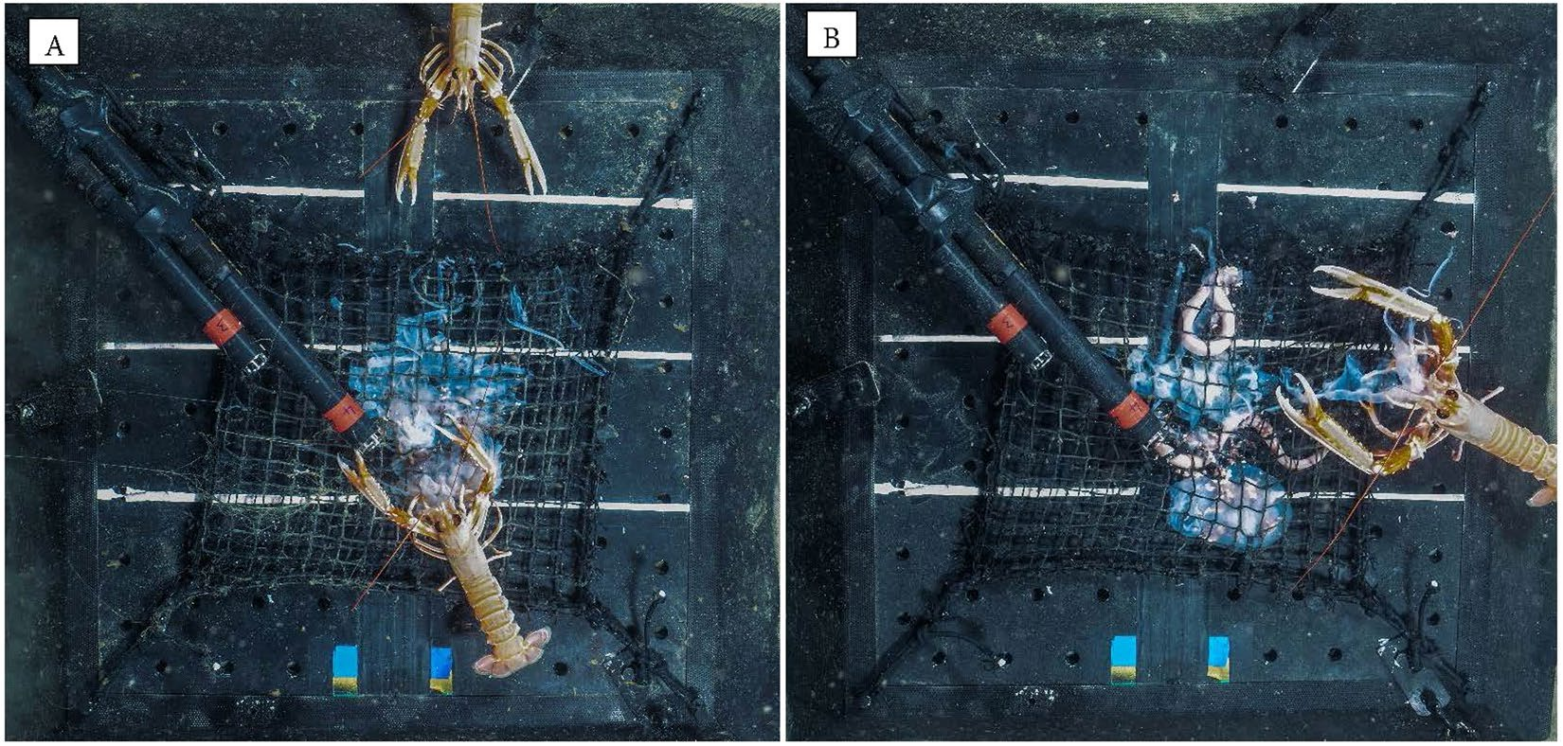
(**A**) Two *Nephrops norvegicus* feeding and approaching the *Periphylla periphylla* bait; and (**B**) an individual *N. norvegicus* removing and feeding on a large portion of gelatinous mesoglea at the bait plate. White lines are spaced 10 cm apart.

A number of different scavenger species consumed the bait in both BUC deployments. Hagfish (*Myxine glutinosa*) always arrived at the bait first (t_arrival_ = 2 minutes and 14 minutes). Other scavengers, such as *M. glutinosa, P. borealis* and *Munida* sp., also consumed the bait, but often declined in abundance when *N. norvegicus* was present (Fig. 1a and b). A maximum of 1 *N. norvegicus* arrived and fed at the bait in deployment 1, while a maximum of 2 *N. norvegicus* were observed in deployment 2 (Fig. 2), with the first *N. norvegicus* arriving 24 (first deployment) and 18 minutes (second deployment) after the lander reached the seafloor. In both deployments *N. norvegicus* consumed a large proportion (>50%) of the bait. *N. norvegicus* first removed what remained of the nutritional gonad tissue, and then continued to feed on the remaining mesoglea tissue. *N. norvegicus* feed during day-light and night hours. It was not possible to detect any influence of time of day on the abundance of *N. norvegicus* (Fig. 1) owing to the low abundance of animals observed, yet this species has been observed to display diurnal patterns of emergence at the depths where we photographed it^22,23^.

The energy intake rate of a 26 g *N. norvegicus* in the Firth of Clyde, Scotland, was found to be approximately 1.97 kJ d^−1^ at a mean temperature of 11 °C^14^. At an *in-situ* temperature of 7.7 °C in Sognefjorden, Q_10_-adjusted energy intake rates would be 1.6 (Q_10_ = 2) to 1.4 (Q_10_ = 3) kJ d^−1^. Therefore, assuming that these energy intake rates are similar to that of the *N. norvegicus* individuals photographed in our study (*N. norvegicus* mass of ~29.8 g estimated from length-mass relationships), 50% of the jellyfish bait consumed in our experiments by *N. norvegicus* (mean energy content of 16.7 kJ g dry weight^−1^) would provide enough energy for a single *N. norvegicus* to survive for 90 (Q_10_ = 2) to 103 days (Q_10_ = 3).

Despite the relatively low energy content of jellyfish material^24^, it is known to be an important food source to a variety of marine predators. For example, the leatherback turtle (*Dermochelys coriacea*) relies upon a diet of low energy-content gelatinous zooplankton^25^, and salps are an important contribution to the diets of bentho-pelagic fish^26^. Commercially exploited invertebrates have been recorded in traps baited with the giant jellyfish *Nemopilema nomurai* ^27^. However, to the best of our knowledge, this is the first study that has directly photographed *N. norvegicus* feeding on a gelatinous organism, and attempted to quantify the importance of jelly-falls as an energy resource to this particular commercially-exploited invertebrate species.

Jellyfish are known to dominate several fjords along the Norwegian coast, including in Lurefjorden and Sognefjorden^7,8^. In Sognefjorden, the abundance of *P. periphylla* is high (100–300 individuals m^−2^), and biomasses here are several orders of magnitude higher than those in the open ocean^7^. This is also true for Lurefjorden, where large pelagic populations of *P. periphylla* contribute (as jelly-falls) to an efficient jelly-pump that can be as important in transporting C and N as the classic phytodetritus pump^5^. Therefore, jellyfish carcass flux data from Lurefjorden was used to estimate how important jellyfish carcasses could be to *N. norvegicus* communities in Sognefjorden. The flux of jelly-fall material transported to the seafloor in Lurefjorden between November 2010 and November 2011 ranged from 12.5 mg to 72.8 mg C m^−2^ d^−1^ or 0.5 to 3.0 kJ m^−2^ d^−1^. The density of *N. norvegicus* in similar environments in other regions of Northern Europe ranges from a minimum of 0.10 to 0.73 individuals m^−2^ ^17^. Therefore, assuming similar seafloor densities for *N. norvegicus* in Sognefjorden, and similar gelatinous carrion flux rates in both Lurefjorden and Sognefjorden, and a conservative consumption of half of the jellyfish, daily jelly-fall fluxes could provide 0.21 to 10.7 times the daily energy requirement for *N. norvegicus* in Sognefjorden. This high energetic contribution from jelly-falls to *N. norvegicus* clearly highlights a potentially important role of gelatinous material in the support of a commercially important species along the Norwegian margin. Even at high *N. norvegicus* densities (0.73 m^−2^) and low gelatinous flux rates (0.5 kJ m^−2^ d^−1^), jelly-falls potentially still provide almost a quarter of the daily energetic demands of *N. norvegicus* populations. Although information on *N. norvegicus* stock sizes have not been collected within fjords, population-size information combined with the type of data presented here may enable the total number of *N. norvegicus* that can be supported by jellyfish carrion to be calculated. This represents valuable information for fisheries management. Such estimates could be further improved with data on *N. norvegicus* foraging patterns, scavenging rates, and the contribution of other food sources to their diets.

This work demonstrates that jelly-falls can provide an important source of nutrition to a commercially-important species in Norway, and suggests that energy transfer pathways from jellyfish to benthic species may become more important in regions where jellyfish blooms presently occur, or are becoming more common (e.g. in numerous Norwegian fjords). There is evidence that the role of fish in some pelagic ecosystems may decline, and by inference, the transport of fish carrion to the seafloor, with increasing jellyfish biomass^8,28–30^. Carrion supply influences deep-sea scavenger community dynamics and changes in the amount of fish carrion reaching the seafloor have been linked to the abundance of deep-sea grenadiers^31^. The findings presented here provide empirical evidence that a loss of energetic resources from fish (and other pelagic animal) carrion to deep-water scavengers could potentially be partially offset by sinking gelatinous material.
